# Investigating the Potential Connection Between Cyberchondria and Vaccine Hesitancy in High School Students

**DOI:** 10.7759/cureus.34218

**Published:** 2023-01-25

**Authors:** Özlem Üzüm, Gülberat Ince, Kayı Eliaçık, Ali Kanık, Ferhan Elmalı, Mehmet Helvacı

**Affiliations:** 1 Department of Pediatric Diseases, University of Health Sciences, Tepecik Training and Research Hospital, Izmir, TUR; 2 Department of Pediatric Diseases, İzmir Katip Çelebi University School of Medicine, İzmir, TUR; 3 Department of Biostatistics, İzmir Katip Çelebi University School of Medicine, Izmir, TUR; 4 Department of Pediatric Diseases, University of Health Sciences, Tepecik Training and Research Hospital, İzmir, TUR

**Keywords:** compulsion, vaccine hesitancy, pandemic, health-related internet use, adolescent

## Abstract

Background: Internet use, which provides the opportunity to access information from anywhere, and anytime, is increasing among adolescents and young adults. In studies examining the effect of technology use in adults, it has been observed that there is a relationship between cyberchondria and vaccine hesitancy. This study aimed to uncover the potential relationship between cyberchondria and vaccine hesitancy in adolescents and to obtain data for combating anti-vaccination in this age group.

Materials and methods: A total of 2.353 high school students were enrolled within the scope of this research. The forms were delivered to the students online and 531 volunteers participated in the survey. The Cyberchondria Severity Scale and Vaccine Hesitancy Scale were used to evaluate the details of the relationship between cyberchondria and vaccine hesitancy levels in adolescents.

Results: The compulsion subscales of the Cyberchondria Severity Scale in males and vaccine repugnance subscales of the Vaccine Hesitancy Scale in females were statistically significantly higher. Cyberchondria and vaccine hesitancy have a strong relationship with each other except benefits and protective value of vaccines subscale of vaccine hesitancy.

Conclusion: Health policymakers should be aware of this relationship and there is a need to develop novel online intervention programs for combating anti-vaccination, particularly among cyberchondriac adolescents who have relatively high vaccination hesitancy, particularly during the pandemic.

## Introduction

The 21st century can be summarized as a time period where dramatic progress has been achieved in curing diseases and developing innovative treatment methods, as well as enhancing preventive healthcare all over the globe [1]. The widespread use of mass media and the availability of information from various sources such as media and the internet increased awareness and, thus, introduced the attitude of seeking health information from these platforms. Currently, a considerable proportion of individuals apply to health institutions with challenging information about themselves [2].

The change in the lifestyle of individuals along with digitalization also changed individuals' behavioral attributes to search for any kind of knowledge. Although technology serves as a useful tool to access data, some people generate excessive distress and anxiety about their health through it [3]. Anxiety about personal health increases in cases of unverified or misinformation received from non-healthcare professionals [4]. The repeated search for information leads to fear and anxiety. This situation is described as a disease called 'cyberchondria', a combination of ‘cyber’ and ‘hypochondria’. The term cyberchondria is the digital version of the ‘hypochondriasis disease’ [4,5]. This can affect the daily productivity and quality of life of individuals, and the treatment demands of these subjects may be a public health burden [6]. These individuals can no longer trust health institutions and, instead, try to find solutions on the internet [7].

People and groups who claim that vaccines are unnecessary and even harmful cause confusion in society about the efficacy and side effects of vaccines by citing different reasons. These individuals are said to have 'vaccine hesitancy' [8]. While hesitancy to vaccination is expected to decrease during severe epidemic periods, unfortunately, vaccine hesitancy has been gaining more and more supporters, especially during the coronavirus disease 2019 (COVID-19) pandemic period [9]. The common usage of social media and other communication channels leverages vaccine hesitancy to reach wider audiences and increase their share [9,10].

The administration of COVID-19 vaccines to adolescents requires obtaining their consent and this became a major issue during the pandemic period. As these individuals will be caregivers and decide on the vaccinations of their children in the future, it is important to impose scientific insight and evaluate their ideas. Additionally, they should have an understanding of the importance of protecting public health. To our knowledge, there is no study in the literature about the possible relationship between cyberchondria and vaccine hesitancy in adolescents. Within the scope of this research, we aimed to elucidate the link between cyberchondria and vaccine hesitancy in adolescents to obtain data for combating anti-vaccination in this age group.

## Materials and methods

Adolescents in selected schools in İzmir, Turkey, between April and May 2022 were included in this study. Cyberchondria Severity Scale (CSS) and Vaccine Hesitancy Scale (VHS) were used to evaluate the relationship between cyberchondria and vaccination hesitancy in adolescents. In addition to the scales, the grade and gender of the adolescents were recorded. Approval for this study was granted by the University of Health Sciences, Tepecik Training and Research Hospital Non-Interventional Research Ethics Committee (Approval number: 2022/02-34, dated February 15, 2022) and the Directorate of National Education (Approval number: 77597247-E-12018877-604.01.02-47840568, dated April 14, 2022) in high school students.

The stratified cluster method was utilized in the selection of the sample population, and the 'school' was used as the sample unit. The power analysis was calculated with a 95% confidence interval (CI), 80% power, and 2% error acceptance, and the overall study population was determined as 2353 students. The questionnaire was applied via draw methodology. Care was taken to have at least one general high school and one vocational high school from each district. A questionnaire was applied to all students who were present on the day of the application and participated in the research voluntarily. Students were informed in advance about the scales and confidentiality. A total of 2.588 high school students in İzmir were invited to participate within the scope of this research. The forms were delivered to the students via the internet and 586 volunteer adolescents accepted to participate in the study and incomplete responders were excluded from the study. Finally, of 586 respondents, 531 were valid (20.5% response rate).

The CSS was defined as a form of anxiety characterized by excessive health research on the Internet by McElroy and Shevlin in 2014, and its Turkish validity and reliability were provided by Uzun and Zencir in 2016. There are 33 questions consisting of a five-point Likert scale (1-Never, 2- Rarely, 3-Sometimes, 4-Frequently, 5- Always). The scale consists of five subscales: (i) compulsion, (ii) distress, (iii) excessiveness, (iv) reassurance, and (v) mistrust of medical professionals. The distrust of doctor subscale is scored inversely. The CSS is not a categorical but a continuous scale and has no cut-off point. The CSS items can be summed to form a total score. The higher scores indicate higher levels of cyberchondria (Cronbach’s alfa=0.94) [11,12].

The VHS was developed by Kılınçarslan et al. in 2020. There are 12 questions consisting of a five-point Likert scale. It has three subscales: (i) benefits and protective value of vaccines, (ii) vaccine repugnance, and (iii) solutions for non-vaccination. The benefits and protective value of vaccine factor items are reverse scored because they consist of statements in favor of the vaccine. Thus, this subscale has also a meaning that higher scores indicate greater vaccine hesitancy. There is no calculated cut-off value. The VHS items can be summed to form a total score. The higher scores indicate higher levels of vaccine hesitancy (Cronbach’s alfa=0.85) [13].

The CSS and VHS scores were examined regarding the grade and gender of the students.

Statistical analysis

Data were evaluated in the IBM SPSS Statistics for Windows, Version 25.0 (Released 2017; IBM Corp., Armonk, New York, United States) statistical package program. Gender distribution and class distribution were given as values and percentages. CSS and VHS scale scores were evaluated with median and standard deviation. Cases were grouped according to these factors and categorical data were compared with Student’s t-test and Chi-square. The relationship between CSS and VHS scores controlling for grade and gender was evaluated by partial correlation coefficient. Pearson correlation was used for the relationship between the scale scores. A p-value <0.05 was considered statistically significant.

## Results

The sample consisted of 216 (40.7%) male and 315 (59.3%) female students. The gender and class distribution are shown in Table 1.

**Table 1:** Gender and grade distribution

Gender	n (%)
Female	315 (59.3)
Male	216 (40.7)
Class/grade	n (%)
9^th^ grade	241 (45.4)
10^th^ grade	172 (32.4)
11^th^ grade	96 (18.1)
12^th^ grade	22 (4.1)

Regarding gender, the compulsion subscale of CSS in males and the vaccine repugnance subscale of VHS in females were statistically significantly higher. The comparison of VHS and CSS scores according to gender is shown in Table 2.

**Table 2:** The evaluation of the students' VHS and CSS scores according to severity and grades VHS: Vaccine Hesitancy Scale; CSS: Cyberchondria Severity Scale p-value˚: Student’s t-test

	Female (n=315/59.3%)	Male (n=216/40.7%)	p-value˚
VHS Total (mean±SD)	29.7±8.3	28.9±8.9	0.290
Benefits and protective value of vaccines	9.6±4.4	9.4±4.5	0.412
Vaccine repugnance	13.5±4.6	12.6±5.1	0.041
Solutions for non-vaccination	6.4±2.5	6.8±3.1	0.112
CSS Total (mean±SD)	74.3±21.6	78.4±26.4	0.062
Compulsion	14.3±6.2	16.4±8.2	0.003
Distress	18.2±6.2	18.7±7.7	0.463
Excessiveness	20.7±7.5	21.1±8.5	0.598
Reassurance	13.2±5.1	14.1±5.9	0.085
Mistrust to medical professional	7.7±3.7	8.2±3.9	0.206

When the bivariate correlations between CSS and VHS were evaluated, it was observed that total and subscale scores of cyberchondria and vaccination hesitancy generally have a strong relationship with each other. The only exception was between the benefits and protective value of the vaccines subscale of VHS and cyberchondria. The details of the correlation analysis are presented in Table 3.

**Table 3:** The correlation analysis of CSS and VHS Scores VHS: Vaccine Hesitancy Scale; CSS: Cyberchondria Severity Scale

	Benefits and protective value of vaccines		Vaccine repugnance		Solutions for non-vaccination		VHS Total	
	r-value	p-value	r-value	p-value	r-value	p-value	r-value	p-value
Compulsion	-0.121	0.005	0.217	<0.001	0.193	<0.001	0.148	0.001
Distress	-0.170	<0.001	0.250	<0.001	0.236	<0.001	0.148	0.002
Excessiveness	-0.211	<0.001	0.228	<0.001	0.201	<0.001	0.128	0.004
Reassurance	-0.195	<0.001	0.217	<0.001	0.188	<0.001	0.077	0.097
Mistrust to medical professional	0.252	<0.001	0.026	<0.001	-0.061	0.160	0.091	0.025
CSS Total	-0.160	<0.001	0.262	<0.001	0.230	<0.001	0.148	0.001

## Discussion

The mass misinformation through online platforms about health issues is one of the most crucial public health problems of the present century and there is limited data about this subject in the literature [14,15]. This study was the first in the literature to examine the relationship between cyberchondria and vaccine hesitancy in the adolescent age group. With regard to gender, male students were more compulsive than females. On the other hand, the vaccine repugnance was significantly higher in females. The findings of the present study elucidated that adolescent cyberchondria may have an interaction with vaccine hesitancy. Especially all subscales with total cyberchondria between vaccine repugnance, solutions for non-vaccination, and total vaccine hesitancy had positive correlations with each other. 

The gender distribution among the volunteer students was similar; however, the class was not homogenous. In our country, the education period is between September and June, and the fourth graders of high school should study more in the last days for the university entrance exam. Due to this, they were not inclined enough to volunteer for this study.

The general vaccine hesitancy, the benefits and protective value of vaccines, and solutions for non-vaccination were not significantly different in terms of gender. However, vaccine repugnance was higher among female students than males. With the pandemic, the anti-vaccination attitude was expected to decrease; on the contrary, vaccine hesitation has been re-discussed [16,17]. Unfortunately, people act with no evidence and several scientific studies expressed negative outcomes on immunization [18]. For this reason, it was thought that with the increase in health literacy, significant changes were expected in anti-vaccine perceptions by increasing people’s ability to access accurate information about vaccines [18]. The higher scores of vaccine hesitancy of females led to the thought that females choose to search the information from unfiltered medical websites.

In terms of gender, the general cyberchondria score and the subscales of distress, excessiveness, reassurance, and mistrust of medical professionals did not show any significant difference. It has been reported that females with higher cyberchondria scores searched for health information more on the internet and felt more anxious than males as females not only feared for themselves but also for their families and friends [19,20]. In addition, the general cyberchondria score was not different in terms of gender in adolescents [20-22]. In our study, there was no difference in CSS between males and females and this was attributed to the fact that the female participants were not mothers yet and did not need to search for information for a special family member such as a child. However, the only significant difference was found in the ‘compulsion’ subscale with higher scores in males. A higher compulsion subscale score elaborated that working productivity was affected due to internet searches for diseases. In the literature, it has been shown that addiction to smartphones and using websites to access information was more common in males. Compulsive behaviors supported obsessive behaviors, and these may disrupt daily work in males [22,23]. Regarding our outcomes, we supposed that the high compulsion level in male students might be related to excessive online searches for information about vaccines, where they were exposed to misinformation.

We observed a strong relationship between cyberchondria and the total score of vaccine hesitancy, subscales, vaccine repugnance, and solutions for non-vaccination. In many studies, it has been shown that the internet was among the determining factors in the decisions of vaccination [16,21]. We assumed that vaccination approaches were shaped by emotional information such as cyberchondria and cognitive information as the perception of the risks of vaccination [4]. The mass amount of information on social media regarding vaccines makes it hard for individuals to judge the reliability of this information [24]. Also, an overload of information triggers cyberchondria and increases false beliefs about vaccine side effects [4]. Instead of obtaining correct information through social media, users are overloaded with existing misinformation [24,25].

While there was a strong correlation between cyberchondria and anti-vaccine total and subscale scores, there were findings that need to be discussed in detail. Although a strong relationship was observed between the reassurance subscale and all subscales of vaccine hesitancy, there was no positive relationship between reassurance and the total score of vaccine hesitancy. The reassurance subscale dimension means a request to consult health professionals after desktop research on the internet in patients with cyberchondria. In addition, it was observed that individuals who were hesitant about vaccination admitted that they could not get sufficient information either from the health professions or that the ministry of health did not provide sufficient information [26,27]. Therefore, it was thought that if the information flow was designed by physicians, this approach might be the most convenient alternative for combating vaccine hesitancy. A negative relationship was detected between cyberchondria, the subscale of anti-vaccine benefits, and the protective value of vaccines. As cyberchondria increase, that is, as anxiety and online medical information research increase, belief in the benefits of the vaccine also increases. COVID-19 pandemic period conditions leveraged individuals searching the disease and commenting on the vaccines.

Limitations and strengths

The results of this study should be interpreted with some limitations. First, the cross-sectional design of this study may have damaged the ability to detect direct causal relationships between the scales. Second, the data was obtained on self-reporting instruments, which may be subject to bias. Third, the contents of the websites that the students checked were not questioned. For this reason, the relation of the contents on the high vaccine repugnance in girls could not be evaluated. Despite these limitations, this is one of few studies to evaluate vaccine hesitancy and cyberchondria in adolescents with highly validated instruments.

## Conclusions

Our findings elucidated an association between vaccine hesitancy and cyberchondria in adolescents. Considering the potential danger of cyberchondria spreading through nonevidence-based rumors, especially during the pandemic, health literacy should be improved and vaccine hesitancy precautions should be taken. By drawing on the findings of the current study and leveraging the power of online media, health policymakers should focus not only on the topic but also on paying more attention to the content to reduce antivaccine misinformation. Future studies should focus on the content of websites that cyberchondriac adolescents frequent or indulge in. Health professionals also have an important role in this regard and they should take responsibility for the success of the mass vaccination program.
